# In Epigenomic Studies, Including Cell-Type Adjustments in Regression Models Can Introduce Multicollinearity, Resulting in Apparent Reversal of Direction of Association

**DOI:** 10.3389/fgene.2019.00816

**Published:** 2019-09-10

**Authors:** Sheila J. Barton, Phillip E. Melton, Philip Titcombe, Robert Murray, Sebastian Rauschert, Karen A. Lillycrop, Rae-Chi Huang, Joanna D. Holbrook, Keith M. Godfrey

**Affiliations:** ^1^MRC Lifecourse Epidemiology Unit, Faculty of Medicine, University of Southampton, Southampton, United Kingdom; ^2^Academic Unit of Human Development and Health, Faculty of Medicine, University of Southampton, Southampton, United Kingdom; ^3^Curtin/UWA Centre for Genetic Origins of Heath and Disease, School of Biomedical Sciences, University of Western Australia, Perth, WA, Australia; ^4^School of Pharmacy and Biomedical Sciences, Curtin University, Perth, WA, Australia; ^5^Telethon Kids Institute, University of Western Australia, Perth, WA, Australia; ^6^Centre for Biological Sciences, Faculty of Natural and Environmental Sciences, University of Southampton, Southampton, United Kingdom; ^7^NIHR Southampton Biomedical Research Centre, University Hospital Southampton NHS Foundation Trust and University of Southampton, Southampton, United Kingdom

**Keywords:** epigenomics, houseman cell-type adjustments, statistical assumptions, multicollinearity, reversal of direction of association, Illumina 450K

## Abstract

**Background:** Association studies of epigenome-wide DNA methylation and disease can inform biological mechanisms. DNA methylation is often measured in peripheral blood, with heterogeneous cell types with different methylation profiles. Influences such as adiposity-associated inflammation can change cell-type proportions, altering measured blood methylation levels. To determine whether associations between loci-specific methylation and outcomes result from cellular heterogeneity, many studies adjust for estimated blood cell proportions, but high correlations between methylation and cell-type proportions could violate the statistical assumption of no multicollinearity. We examined these assumptions in a population-based study.

**Methods:** *CDKN2A* promoter CpG methylation was measured in peripheral blood from 812 adolescents aged 17 years (Western Australian Pregnancy Cohort Study). Log_e_ adolescent BMI was used as the outcome in a regression analysis with DNA methylation as predictor, adjusting for age/sex. Further regression analyses additionally adjusted for estimated cell-type proportions using the reference-based Houseman method, and simulations modeled the effects of varying levels of correlation between cell proportions and methylation. Correlations between estimated cell proportions and CpG methylation from Illumina 450K were measured.

**Results:** Lower DNA methylation was associated with higher BMI when cell-type adjustment was not included; for CpG4, β = −0.004 log_e_BMI/%methylation (95% CI −0.0065, −0.001; *p* = 0.003). The direction of association reversed when adjustment for six cell types was made; for CpG4, β = 0.004 log_e_BMI/%methylation (−0.0002, 0.0089; *p* = 0.06). Correlations between CpG methylation and cell-type proportions were high, and variance inflation factors (VIFs) were extremely high (25 to 113.7). Granulocyte count was correlated with BMI, and removing granulocytes from the regression model reduced all VIFs to <3.1, with persistence of a positive association between methylation and BMI [CpG4 β = 0.004 log_e_BMI/%methylation (−0.0002, 0.0088; *p* = 0.06)]. Simulations supported major effects of multicollinearity on regression results.

**Conclusions:** Where cell types are highly correlated with other covariates in regression models, the statistical assumption of no multicollinearity may be violated. This can result in reversal of direction of association, particularly when examining associations with phenotypes related to inflammation, as CpG methylation may associate with changes in cell-type proportions. Removing predictors with high correlations from regression models may remove the multicollinearity. However, this might hinder biological interpretability.

## Introduction

Association studies of DNA methylation and human disease can yield insights into biological mechanisms. Methylation is often measured in peripheral blood; however, blood is a heterogeneous collection of cell types, each with a different methylation profile. Inter-individual differences in DNA methylation may therefore be driven at least in part by differences in cell populations within the measured tissue type. Distinguishing between the “intrinsic methylation signal,” i.e., that independent of cellular heterogeneity and that caused by differential mixtures of cell types, is especially problematic when studying methylation changes associated with disorders exemplified by chronic inflammation (Holbrook et al., 2017). For example, in obesity and type 2 diabetes, inflammatory responses can change cell proportions within blood, altering methylation levels. There has been much debate in recent years (Houseman et al., 2015; Horvath et al., 2016; Holbrook et al., 2017; Quach et al., 2017) as to whether proportion of cell types is a confounder to be removed from an analysis, or a source of valuable information about disease etiology and comorbidity.

To identify the variation in loci-specific DNA methylation associated with disease phenotype rather than variation related to differences in cell populations, it is common to adjust for estimated blood cell proportions during statistical analysis. The reference-based Houseman method (Houseman et al., 2012) uses data from 100 CpG sites shown to be differentially methylated between FACS-sorted leukocyte subgroups and measured on Infinium Beadarray (the sites contained within the HumanMethylation27, HumanMethylation450 and EPIC arrays) (Illumina Inc., San Diego, CA) methylation platform to estimate the proportion of leukocyte subtypes in unfractionated whole blood. The advantage of this method is that it does not require sorted cells but relies on the prior reference data to estimate proportions in the sample under study. The reference set was based on 46 white blood cell samples, with an additional 27 whole blood samples used as controls to estimate batch effects. Hitherto, there have however been no formal examinations of whether including cell-type proportions in regression equations might violate regression assumptions and lead to challenges in interpreting findings. This study therefore examined for evidence of multicollinearity in a population-based epigenetic study, alongside simulations to model the effects of varying levels of correlation between cell proportions and methylation.

## Materials and Methods

### The Raine Study: Participants

The Raine Study (Straker et al., 2017) enrolled pregnant women ≤18th week of gestation (1989–1991) (*N* = 2,900) through the antenatal clinic at King Edward Memorial Hospital and nearby private clinics in Perth, Western Australia. Detailed clinical assessments were performed at birth. Birth information (including birth weight and height) was obtained from midwife records. The children were followed up at multiple time points, including at 17 years of age (Generation 2) when physical assessments including weight, height, and skin fold assessments were performed as described previously (Huang et al., 2015a). Socioeconomic status was assessed by maternal education. Maternal weight and height were measured by a trained midwife at 18 weeks’ gestation. Early pregnancy weight was obtained at recruitment around 18 weeks’ gestation. Gestational age was based on the date of the last menstrual period unless there was discordance with ultrasound biometry at the dating scan. Characteristics of the Generation 2 Raine Study participants are shown in **Supplementary Table 1**.

### Ethics Approval and Consent to Participate

The Human Ethics Committees (King Edward Memorial Hospital and/or Princess Margaret Hospital) approved all protocols (RA/4/1/6613). Informed, written consent to participate in the study was obtained from the mother of each child at enrolment and at each subsequent follow-up.

### Pyrosequencing

The levels of DNA methylation in the promoter of the long non-coding RNA ANRIL that is encoded within the *CDKN2A* gene locus were measured in peripheral blood from Generation 2 adolescents from the Raine Study by sodium bisulfite pyrosequencing, as previously described (Murray et al., 2016; Lillycrop et al., 2017). This region of *CDKN2A* (genomic locations listed in **Supplementary Table 2**) is not covered by Illumina 27K, 450K, or EPIC(850K) methylation arrays and was first identified through a genome-wide screen of methylation differences at birth associated with % fat mass of children aged 6 years in the UK Southampton Women’s Survey, along with similar findings in birth tissues from ethnically diverse neonates and in adipose tissue from adults (Lillycrop et al., 2017). Further studies also showed an association between the methylation status of CpGs 4–9 within this DMR in peripheral blood of 17-year-old adolescents with measures of concurrent adiposity in the Raine study (Lillycrop et al., 2017).

### Houseman Cell-Type Estimation From 450K Methylation Array Data

DNA was extracted from whole blood samples obtained at 17-year-old (Raine Study Generation 2) follow-up. Bisulfite conversion was prepared from whole blood cells by standard phenol:chloroform extraction and ethanol precipitation. Processing of the Illumina Infinium HumanMethylation450 BeadChips was carried out by the Centre for Molecular Medicine and Therapeutics (CMMT) (http://www.cmmt.ubc.ca) for 1,192 samples and 58 technical replicates. DNA methylation beta-values were normalized using Beta-mixture quantile dilation (BMIQ) as described by Teschendorff et al. (2013). Three samples identified as outliers and one sample for sex inconsistency during quality control were excluded. Cell-type correction was determined using the reference-based Houseman method in the minfi package (van Iterson et al., 2014) using R. This method estimates the relative proportions of white blood cell subtypes [six were measured in our study: CD4+ T-lymphocytes, CD8+ T-lymphocytes, NK (natural killer) cells, B-lymphocytes, monocytes, and granulocytes], based on a standard reference population. Spearman correlations were calculated between each estimated cell type and CpG methylation.

### Statistical Analysis

Statistical analysis was carried out using Stata (Statacorp) versions 11.2 to 14.2 and R version 3.3. Histograms of continuous variables were plotted to check for normality. The distribution of BMI in this cohort was positively skewed and therefore transformed using a log_e_ transformation. Regression models were built using adolescent’s log_e_ BMI measurement at 17 years (Generation 2) as the outcome and CpG methylation as the predictor. Models were adjusted for adolescent’s sex and exact age at measurement. Results are presented as regression coefficients (β), which represent the (mean) change in outcome (log_e_BMI) for a one unit (%) increase in methylation, with their standard errors, 95% confidence intervals (CIs) and associated *p* values. Further regression analysis was performed adjusting for six estimated cell-type proportions (CD8 T cells, CD4 T cells, NK cells, B cells, monocytes, and granulocytes) to account for differences in cellular heterogeneity in blood, in addition to age and sex.

The correlations between observed DNA methylation values at the nine CpG dinucleotides and six estimated Houseman cell types were examined using Pearson correlation coefficients (see **Supplementary Table 3**). As these correlations were found to be high, to assess possible violation of regression assumptions (Gujarati and Porter, 2009; Barton et al., 2013), variance inflation factors (VIFs) were calculated in R using the car (Companion to Applied Regression) package (Weisberg and Fox, 2011) for all coefficients in regression equations for each CpG (see **Supplementary Table 4**). VIFs measure how much the variances of the regression coefficients (βs) are inflated compared to no correlation between predictor variables. The percentage of variance explained by the other independent variables can be calculated using the formula (1 − (1/VIF))*100. A VIF of 5 or less, corresponding to 80% of the variance explained by other independent variables, is generally thought to be acceptable (Gujarati and Porter, 2009). Regression models were re-run excluding the cell type with the highest VIF (granulocytes) in order to reduce multicollinearity. In addition principal components (PCs) of the six estimated cell-type proportions (CD8 T cells, CD4 T cells, NK cells, B cells, monocytes, and granulocytes) were calculated using the “prcomp” package in R, and models were run including the first two PCs instead of all six estimated cell types, in order to reduce multicollinearity. A stepwise approach was also used, regressing out each cell type variable from the CpG methylation measurements to minimize multicollinearity at each step, and then the methylation residuals were regressed against log_e_ BMI. To further investigate this relationship, we conducted Spearman correlation between five of the six estimated cell counts (CD8T, CD4, B cells, monocytes, and granulocytes) with the CpG probes from the Illumina Infinium HumanMethylation450 array in R.

A simulation was constructed to estimate how high correlation between predictors could be without inducing multicollinearity into the regression. Datasets were constructed by sampling from a Normal distribution with the same mean and standard deviation as log_e_ BMI, CpG4, and granulocyte counts, specifying correlations between log_e_ BMI, CpG4, and granulocytes to be the same correlations observed in our study. One thousand regressions were run using this simulated data, and we measured how often the regression coefficient for CpG4 was observed to be positive. The correlation between CpG4 and granulocytes was then changed from a high correlation (*r* = −0.783, as in our original data) to a mid-range correlation (*r* = −0.5) and a low correlation (*r* = −0.25), and the simulations were re run 1,000 times for each scenario.

## Results

Lower *CDKN2A* DNA methylation was associated with higher BMI in the Raine Study when cell-type adjustment was not included for CpGs 4 to 9 (Lillycrop et al., 2017) (**Table 1**, rows 1–4). However, when adjustment for estimated proportions of CD8 T cells, CD4 T cells, B cells, natural killer cells, monocytes, and granulocytes (estimated using the Houseman method) were included in the regression model, the direction of association was reversed for all of these CpGs (4 to 9), with CpGs 4 and 8 being no longer significantly associated with log_e_ BMI (**Table 1**, rows 5–7). For all CpGs except CpG2, the standard error of regression coefficients increased when cell-type adjustments were included in the model.

**Table 1:** Regression results for Log_e_ BMI with DNA methylation % as predictor adjusted for age and sex: i) without Houseman cell-type adjustment; ii) with six Houseman cell-type adjustments; iii) with Houseman cell-type adjustments but excluding granulocytes; iv) with the first two principal components of six Houseman cell-type adjustments; v) cell-type adjustments residuals, each cell type individually; vi) cell-type adjustment residuals all cell types together. se, standard error.

Row	CpG	CpG1	CpG2	CpG3	CpG4	CpG5	CpG6	CpG7	CpG8	CpG9	
**1**	**N**	780	758	723	812	790	778	740	801	760	
**2**	**β**	0	0.004	−0.004	−0.004	−0.003	−0.004	−0.004	−0.004	−0.004	log_e_ BMI
**3**	**Se**	0.001	0.0029	0.0019	0.001	0.001	0.0012	0.001	0.0012	0.0012	
**4**	***P*** **value**	0.915	0.98	0.06	0.003	0.012	0.003	0.008	0.001	0.006	(Age and sex adjusted only)
**5**	**β**	0.005	0.003	0.001	0.004	0.006	0.005	0.007	0.005	0.006	log_e_ BMI
**6**	**se**	0.0017	0.0014	0.0024	0.0023	0.0022	0.0022	0.0024	0.0025	0.0024	
**7**	***P*** **value**	0.01	0.041	0.608	0.061	0.007	0.028	0.005	0.063	0.009	(Age, sex, and Houseman cell-type adjustments)
**8**	**β**	0.005	0.003	0.001	0.004	0.006	0.005	0.007	0.005	0.006	log_e_ BMI
**9**	**se**	0.0017	0.0010	0.0024	0.0023	0.0022	0.0022	0.0024	0.0025	0.0025	
**10**	***P*** **value**	0.010	0.041	0.610	0.064	0.007	0.031	0.004	0.062	0.009	(Age, sex, and Houseman cell-type adjustments without granulocytes)
**11**	**β**	0.005	0.003	0.001	0.004	0.006	0.005	0.007	0.005	0.006	log_e_ BMI
**12**	**se**	0.0017	0.0014	0.0024	0.0023	0.0022	0.0022	0.0024	0.0025	0.0024	
**13**	***P*** **value**	0.01	0.041	0.608	0.061	0.007	0.028	0.005	0.063	0.009	(Age, sex, and principal components 1 and 2 of Houseman cell-type adjustments)
**14**	**β**	0.096	0.062	0.014	0.043	0.067	0.049	0.064	0.048	0.089	log_e_ BMI
**15**	**se**	0.0436	0.0361	0.0598	0.0536	0.0524	0.0508	0.0553	0.0584	0.0564	
**16**	***P*** **value**	0.028	0.087	0.820	0.427	0.199	0.331	0.246	0.415	0.115	(Age, sex, and Houseman cell-type residuals one at a time)
**17**	**β**	0.121	0.084	0.045	0.109	0.147	0.120	0.173	0.132	0.172	log_e_ BMI
**18**	**se**	0.0446	0.0368	0.0619	0.0592	0.0578	0.0568	0.0620	0.0664	0.0640	
**19**	***P*** **value**	0.007	0.022	0.468	0.065	0.011	0.035	0.005	0.047	0.007	(Age, sex, and Houseman cell-type residuals all at the same time)

Correlations between *CDKN2A* CpG methylation and cell-type proportions were found to be high (**Supplementary Table 3**), and the outcome, log_e_ BMI, was also correlated with cell-type proportions, particularly granulocytes (*r* = 0.16, **Supplementary Table 5**). As log_e_ BMI is the outcome in the regression, this will not result in multicollinearity and will not alter the regression coefficients of the predictors. **Table 2** shows correlations between CpG4 and estimated cell proportions. Therefore, VIFs were calculated for all predictors in the regression models (**Supplementary Table 4**); VIFs, 1/VIF (tolerance), and percentage variance explained by the other independent variables for the model including CpG4 are shown in **Table 3**.

**Table 2:** Pearson correlation between CpG4 and estimated cell proportions.

CpG4	CD8 T cells	CD4 T cells	NK cells	B cells	Monocytes	Granulocytes
Pearson correlation	.611^**^	.553^**^	.371^**^	.215^**^	–.402^**^	–.783^**^
Significance (two-tailed)	<2.2 × 10^−16^	<2.2 × 10^−16^	<2.2 × 10^−16^	8.19 × 10^−13^	<2.2 × 10^−16^	<2.2 × 10^−16^
*N*	1081	1081	1081	1081	1081	1081

**Table 3:** Variance inflation factors (VIFs), 1/VIF, and percentage variance explained by the other independent variables, for the model including CpG4. The left-hand side of **Table 3** shows VIFs, 1/VIF, and percentage variance explained by the other independent variables for the model including six Houseman cell types; the right-hand side of **Table 3** shows results for the model including five Houseman cell types but omitting granulocytes.

log_e_ BMI	VIFs	1/VIF	% Variance explained	VIFs without granulocytes	1/VIF	% Variance explained
**Sex**	1.20	0.831	16.87	1.19	0.84	16.11
**Age**	1.03	0.975	2.53	1.02	0.98	1.64
**CpG4**	3.17	0.316	68.43	3.13	0.32	68.06
**CD8 T cells**	24.95	0.040	95.99	1.83	0.55	45.35
**CD4 T cells**	61.21	0.016	98.37	2.09	0.48	52.06
**NK cells**	30.26	0.033	96.70	2.02	0.49	50.57
**B cells**	14.67	0.068	93.18	1.33	0.75	25.08
**Monocytes**	11.32	0.088	91.17	1.50	0.67	33.19
**Granulocytes**	113.71	0.009	99.12	NA	NA	NA

**Table 3** shows that some VIFs were found to be extremely high, particularly the VIFs for granulocytes (113.71) and for CD4 T cells (61.21), and that these cell types did not contribute much to the models as most of their variance was explained by other independent variables in the model. Removing one cell type with the largest VIF (granulocytes) from the regression model reduced all VIFs to acceptable levels (< 3.13), as shown on the right-hand side of **Table 3**. However, omitting granulocytes from the regression did not reduce multicollinearity sufficiently to allow accurate estimation of regression coefficients for CpG methylation as shown in **Table 1**, rows 8–10. The regression coefficients of CpGs 4–9 are still negative and the standard errors are inflated. Rows 11–13 of **Table 1** show that including the first two PCs of all six Houseman cell-type adjustments does not reduce multicollinearity either. This was because correlations between the first two PCs and CpGs were very high (for PCV1: *r* = −0.329 to −0.751, *p* < 2.22 × 10^−13^) as shown in **Table 4**, and this correlation induced further multicollinearity into the regression.

**Table 4:** Pearson correlation between CpGs and first two principal components (PCs) of six Houseman cell-type adjustments.

		PC1	PC2
**CpG1**	Pearson correlation	−.470**	.258**
	Significance (two-tailed)	< 2.22 × 10^−13^	< 2.22 × 10^−13^
	*N*	781	781
**CpG2**	Pearson correlation	−.329**	.297**
	Significance (two-tailed)	< 2.22 × 10^−13^	< 2.22 × 10^−13^
	*N*	759	759
**CpG3**	Pearson correlation	−.493**	.311**
	Significance (two-tailed)	< 2.22 × 10^−13^	< 2.22 × 10^−13^
	*N*	724	724
**CpG4**	Pearson correlation	−.671**	.447**
	Significance (two-tailed)	< 2.22 × 10^−13^	< 2.22 × 10^−13^
	*N*	813	813
**CpG5**	Pearson correlation	−.653**	.464**
	Significance (two-tailed)	< 2.22 × 10^−13^	< 2.22 × 10^−13^
	*N*	791	791
**CpG6**	Pearson correlation	−.700**	.456**
	Significance (two-tailed)	< 2.22 × 10^−13^	< 2.22 × 10^−13^
	*N*	778	778
**CpG7**	Pearson correlation	−.645**	.496**
	Significance (two-tailed)	< 2.22 × 10^−13^	< 2.22 × 10^−13^
	*N*	741	741
**CpG8**	Pearson correlation	−.751**	.415**
	Significance (two-tailed)	< 2.22 × 10^−13^	< 2.22 × 10^−13^
	*N*	802	802
**CpG9**	Pearson correlation	−.727**	.436**
	Significance (two-tailed)	< 2.22 × 10^−13^	< 2.22 × 10^−13^
	*N*	761	761

**Figure 1:**
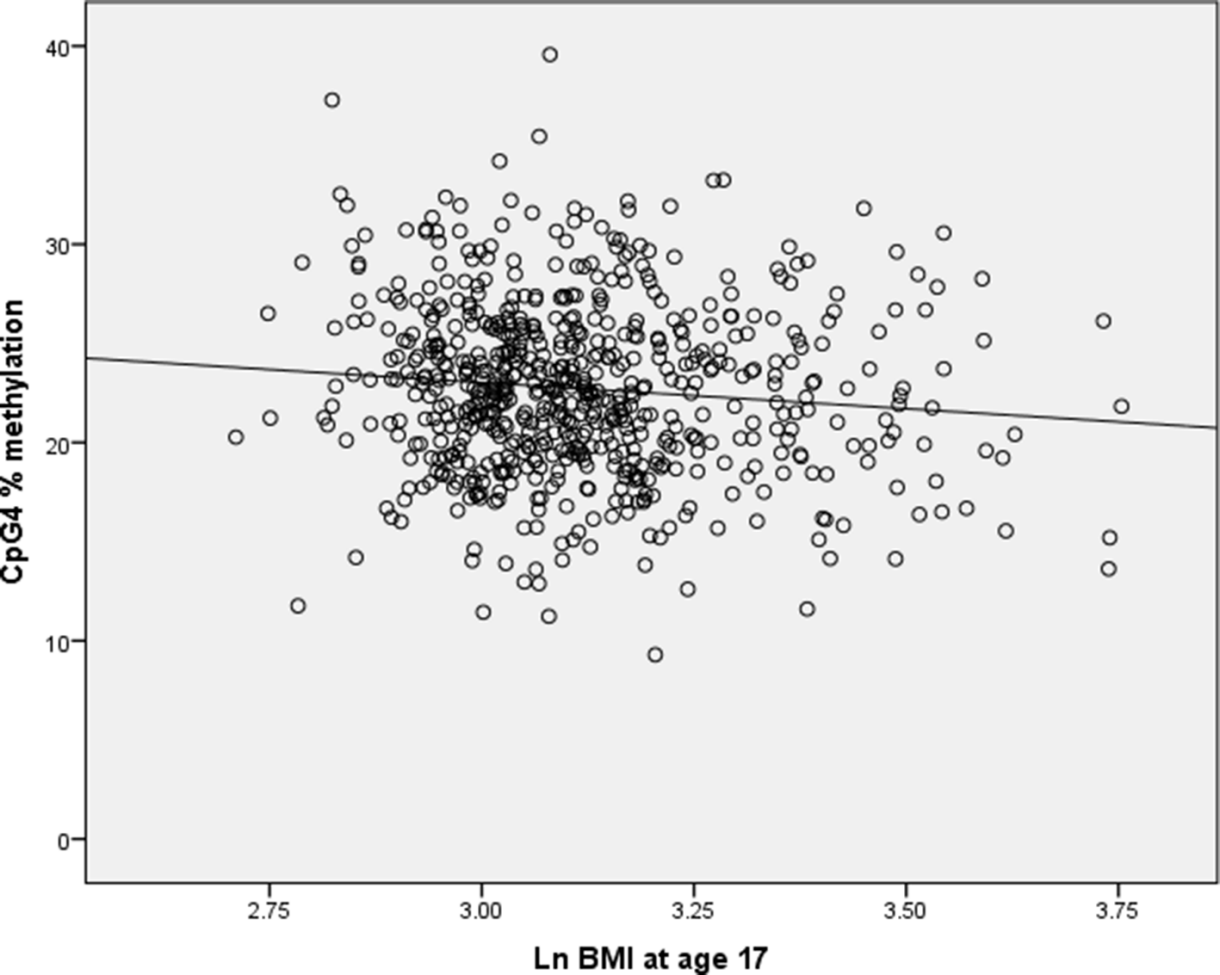
Scatterplot of CpG4% methylation against log_e_ BMI at age 17 in the Raine cohort.

Simulation results showed that when a simulated granulocyte variable was not included in the models, 0.5% of the regression coefficients for CpG4 were positive. As the correlation between CpG4 methylation and log_e_ BMI is negative, we would expect the regression coefficient of CpG4 methylation vs. BMI to be negative (see **Figure 1**). When granulocyte counts were included in the simulation models with a correlation coefficient of −0.783 with CpG4, 83% of the regression coefficients for CpG4 were positive. For correlation between CpG4 and granulocyte count of *r* = −0.5, 27.7% of the regression coefficients for CpG4 were positive, and for a correlation of *r* = −0.25, 6% of the regression coefficients for CpG4 were positive. This indicates that very high correlations between predictors are likely to cause multicollinearity and apparent reversal of direction of effect.

Examining the wider potential for collinearity effects relating to blood cell proportions, we examined the correlations between the DNA methylation status of CpG sites on the HumanMethylation 450K BeadChip, a commonly used platform for EWAS studies, and Houseman cell-type estimates in 1,192 samples. 11,193 (2.36%) CpGs on this array were highly correlated (*r*_s_ ≥ |0.700|) with the peripheral blood granulocyte count; 6,023 (1.27%) CpGs were highly correlated with CD4 T cell count; 146 (0.03%) were highly correlated with B cell count; 25 (0.0053%) were highly correlated with CD8 T cell count; and 1 CpG (0.00021%) was highly correlated with monocyte count (cg13430807 in MTMR11). Estimated natural killer cell proportions were very low in these data and therefore correlations between NK cells and CpG methylation were not calculated. CpGs that are highly correlated with cell-type estimates are likely to cause multicollinearity when included as adjustments in regressions with these CpGs as predictors. The 50 CpGs that were most strongly correlated with granulocytes, CD4 T cells, B cells, and the 25 CpGs that were correlated with CD8 T cells (*r*_s_ ≥ |0.700|) are listed in **Supplementary Tables 6**, **7**, **8**, and **9**.

## Discussion

Our findings show that including Houseman cell-type adjustments in regression models can introduce multicollinearity into the models and lead to unstable estimates and inflated standard errors of regression coefficients. This resulted in models where both the direction of effect of predictors and the significance of regression coefficients changed. It is important that multicollinearity in regression equations is detected and steps are taken to minimize it, to ensure regression coefficients, and subsequent interpretations, are correct.

Multicollinearity (also referred to as collinearity) occurs when one predictor in a multiple regression model can be linearly predicted from the other predictors with a substantial degree of accuracy. Thus, the method used to derive cell-type proportions in our study assumes that there are only six cell types present in blood and therefore the proportions should add to 1 (Houseman et al., 2012). Therefore, in theory, including all six cell types as predictors in a regression should result in one of the cell-type coefficients being inestimable and a warning message generated by the statistical software. In practice, due to noise and error in the method, the six estimated cell types often sum to greater than 1 and the warning message is not generated. In theory, omitting one of these six cell types for the regression should solve this problem, but typically it is the lowest estimated cell type that is omitted and this may not resolve the issue. In our study, as the remaining cell types were still highly correlated with CpG methylation, the issue of multicollinearity remained.

Multicollinearity can also occur when two or more predictors in a regression model are highly correlated, as it results in the same or very similar information being entered into the model two or more times (Gujarati and Porter, 2009). One of the assumptions of the classical linear regression model is that there is no multicollinearity among the predictors included in the regression model. If this assumption is not met, estimates of regression coefficients of the highly correlated predictor variables are difficult to determine and their standard errors are inflated. Multicollinearity can cause the regression coefficients to change signs, reversing the apparent direction of effect of a predictor. However, multicollinearity does not affect model fit or the ability of the model to predict and so need not necessarily be a problem (Gujarati and Porter, 2009). It is common to have some degree of multicollinearity in regression models and mild to moderate multicollinearity can often be tolerated. However, when multicollinearity is severe and estimates of regression coefficients are required for inference, this can be a major issue. In the epigenetic analyses that we undertook, we found that VIFs were particularly high for two components of cell-type adjustments (granulocytes and CD4 T cells), which are frequently used as adjustment covariates in population DNA methylation studies. Omitting granulocytes and CD4 T cells either individually or together did not attenuate multicollinearity sufficiently to allow accurate estimates of CpG regression coefficients as the remaining cell types were also highly correlated with CpG methylation.

High correlation between cell-type estimates and the outcome of interest (log_e_BMI in our study) will not lead to multicollinearity as they are on opposite sides of the regression equation. High correlation between the cell-type estimates themselves could cause multicollinearity in the regression, but this will only be an issue if an estimate of the regression coefficient of the Houseman cell type is of interest. However, outcomes such as height that are not related to cell type are less likely to result in multicollinearity in the regressions as it is less likely that cell types will be correlated to CpG sites associated with height.

We also observed that proportions of some cell types were low in our data, for example, natural killer cells. Including this cell type as a predictor will not add much information to the model but also has the potential to cause multicollinearity if the correlation between the cell-type estimate and CpG methylation is high (see **Supplementary Table 3**).

The main question to address is not whether multicollinearity is present or absent in a regression equation, but the extent of possible multicollinearity and whether it is affecting the estimates of regression coefficients of interest. Multicollinearity is not, in general, easy to detect. It is always good practice to plot each predictor variable against each other and against the outcome to look for high levels of linear correlation between variables. This can be a good indication that multicollinearity is likely to be an issue. VIFs can also be calculated to indicate multicollinearity as described in the Materials and Methods section, and condition indices (Gujarati and Porter, 2009), though more complicated to interpret, can also be useful. However, unfortunately, there are no hard and fast rules about how much multicollinearity can be tolerated using these or other measures. The most reliable methods, which are somewhat subjective, tend to be i) looking for regression models where the variance explained by the model (*R*^2^) is high and yet few or none of the predictors are significantly associated with the outcome, ii) unexpected changes in regression coefficients and standard errors when small changes are made to the model, and/or iii) looking for high correlation between predictor variables.

There are ways to deal with multicollinearity, but none are entirely satisfactory. One possible approach to reduce multicollinearity is by calculating PCs among the cell-type proportions and using some of the PCs as predictors instead of the individual estimates of cell type proportions in the regression models. Although this reduces multicollinearity between the estimates of cell type proportion, in our data, it did not resolve the problem of collinearity between CpG4 and cell-type proportion as the first two PCs were also highly correlated with CpG methylation and therefore induced further multicollinearity. Another approach is to individually regress out cell-type adjustments from the methylation measurements to minimize multicollinearity at each step, and then to regress the residuals against outcome (log_e_ BMI). This was done regressing out each cell type individually (**Table 1**, rows 14–16) and regressing out all cell types at the same time (**Table 1**, rows 17–19). Neither approach resolved the problem in our data due to the complex relationship between cell-type estimates and difficulty in establishing a sequence of importance for the cell-type estimates (Graham, 2003).

The data used for our study measures both CpG methylation and BMI at age 17 in the Generation 2 Raine study participants, and this makes it difficult to assign causality. However, in some circumstances, it might be possible to use CpG methylation as the outcome and log_e_ BMI as a predictor, but as log_e_ BMI is also correlated with five out of the six Houseman cell types (see **Supplementary Table 5**), multicollinearity may still be present in the regressions in our study. It can also be statistically problematic using methylation measurements as an outcome in a regression because the distributions of these measurements are rarely normally or approximately normally distributed. Transformation to *M* values instead of beta values (Du et al., 2010) will not be beneficial as the correlation between CpG methylation and cell types is still very high when *M* values are used.

It is therefore very hard to detect whether multicollinearity could be an issue in published papers where results of regression equations are only presented fully adjusted and it is suspected that many of the predictors could be highly correlated. Even authors who correctly try and determine the extent of multicollinearity using VIFs can still violate the assumption of no multicollinearity if unadjusted regressions, scatterplots, or correlation coefficients of all predictors and outcome are not presented (Garcia et al., 2017). Many studies on DNA methylation and obesity have been carried out to date (de Mello et al., 2014; Garcia-Cardona et al., 2014; Huang et al., 2015b; Holbrook et al., 2017; Lillycrop et al., 2017; Quach et al., 2017), and most of these studies adjust for cell types. Interpretation of the results of these studies is problematic if there is likely to be severe multicollinearity between predictors, i.e., if there is high correlation between CpG methylation and cell-type proportions.

Some researchers might consider the absence of sorting for cell type at sample collection, as is usual in population-based studies of any size with phenotypic information, as a limitation of our study; this precludes direct analyses of blood cell-type-specific methylation, but the absence of such information does not negate our findings, particularly the simulation data. Another limitation is that the cross-sectional nature of our study in participants with established excess adiposity is likely to have increased the chance of some of the cell types being highly correlated with CpG methylation, driven by adiposity effects on inflammatory processes. Such correlation may be less likely in longitudinal studies in which excess adiposity was not present at baseline. Where a strong correlation between cell types and CpG methylation is present, this could be of benefit for formulating therapeutic targets, providing the assumptions required for linear regression are met.

Strengths of this paper are that we show, using an explanation readily accessible to readers, that adding highly collinear variables such as cell-type adjustments into regression equations can induce multicollinearity, which makes interpretation of regression coefficients problematic. We suggest practical methods to detect multicollinearity and explain metrics (e.g., VIFs) commonly used to detect multicollinearity. We have also investigated correlations between Houseman estimates of cell type for our data and CpG methylation in the Illumina 450K Human Methylation platform to give researchers an idea of how widespread this problem is likely to be. We have run simulations to indicate how much correlation between variables can be tolerated between predictors in a regression without causing multicollinearity, resulting in difficulty in interpreting regression coefficients. If these recommendations are taken on board, this has the potential to improve the quality and clarity of published papers and resolve possible ambiguity between different research groups where the directions of effect are found to be in the opposite direction.

The limitations of these recommendations are that there is much debate in the statistical community about the most useful way to detect the extent of collinearity and there are no hard and fast rules for the metrics suggested such as VIFs.

## Conclusion

Houseman cell-type adjustment is an important feature of DNA methylation research as it allows correction for cell type when using peripheral blood. However, if estimated cell types are highly correlated with other covariates in regression models, the statistical assumption of no multicollinearity may be violated. This can result in apparent reversal of direction of association or loss of statistical significance for predictors. This is particularly important to consider when understanding associations with phenotypes related to inflammation, as CpG methylation may then be associated with changes in cell-type proportions. Assessment of possible multicollinearity and taking steps to minimize it is essential to ensure regression coefficients, and subsequent interpretations, are correct.

## Data Availability

The datasets used and/or analyzed during the current study are available by application to the Raine Study Executive Committee on reasonable request.

## Ethics Statement

The Human Ethics Committees (King Edward Memorial Hospital and/or Princess Margaret Hospital) approved all protocols (RA/4/1/6613). Informed, written consent to participate in the study was obtained from the mother of each child at enrollment and at each subsequent follow-up.

## Author Contributions

SB, JH, KG, and KL conceived and designed the study. SB, PM, PT, and SR carried out the statistical analysis. R-CH and RM performed the laboratory work and analysis. SB, PM, and KG wrote the manuscript in conjunction with R-CH, JH, PT, SR, KL, and RM. All authors read and approved the final manuscript.

## Funding

The DNA methylation work was supported by NHMRC grant 1059711. The Generation 2 (17-year) follow-up was supported by National Health and Medical Research Council Program grant (ID353514) and Project grant (ID403981). R-CH is supported by NHMRC Fellowships (grant numbers 1053384 and 1042255, respectively). SR is supported by National Health and Medical Research Council EU grant (1142858) and the Department of Health, Western Australia Future Health fund in connection with the European Union’s Horizon2020 grant 733206. KG is supported by the UK Medical Research Council (MC_UU_12011/4), the National Institute for Health Research (as an NIHR Senior Investigator) (NF-SI-0515-10042), and the European Union’s Erasmus+ Capacity-Building ENeASEA Project. KL and KG are supported by the National Institute for Health Research through the NIHR Southampton Biomedical Research Centre and by the European Union’s Seventh Framework Programme (FP7/2007-2013), project EarlyNutrition under grant agreement n°289346.

## Conflict of Interest Statement

KG has received reimbursement for speaking at conferences sponsored by companies selling nutritional and pharmaceutical products. Some of the research groups involved in this work are part of an academic consortium that has received research funding from Abbott Nutrition, Nestec, and Danone. The remaining authors declare that the research was conducted in the absence of any commercial or financial relationships that could be construed as a potential conflict of interest.

## Abbreviations

DMR, differentially methylated region; DNA, deoxyribonucleic acid; BMI, body mass index; CAR, companion to applied regression; CI, confidence intervals; CpG, 5′-C-phosphate-G-3′; FACS, fluorescence-activated cell sorting; NK cells, natural killer cells; PC, principal components; RNA, ribonucleic acid; VIF, variance inflation factor
