# Supplementary material for: In Epigenomic Studies, Including Cell-Type Adjustments in Regression Models Can Introduce Multicollinearity, Resulting in Apparent Reversal of Direction of Association
Source: Front Genet. 2019 Sep 10;10:816. doi: 10.3389/fgene.2019.00816 (PMC6746958; doi:10.3389/fgene.2019.00816)
Supplement: Supplementary file 1 [file Table_1.docx]

| **Characteristic** | **Group/number** | **% or median (inter-quartile range)** |
| --- | --- | --- |
| **Mother** |  |  |
| **Qualification level** | High school only | 51.60% |
|  | Trade Certificate or apprenticeship | 8.40% |
|  | Professional Registration | 8.80% |
|  | Diploma | 16.00% |
|  | University Degree | 10.00% |
|  | Other | 5.20% |
| **Smoker During Pregnancy** | No | 82.80% |
|  | Yes | 17.20% |
| **Parity** | Primiparous | 49.40% |
|  | Multiparous | 50.60% |
| **Age at birth of child (years)** | 2804 | 28 (23 to 32) |
| **Early pregnancy BMI** | 2803 | 21.3 (19.6 to 23.7) |
| **Offspring** |  |  |
| **Sex** | Male | 50.70% |
|  | Female | 49.30% |
| **Birthweight (g)** | 2858 | 3345 (2984 to 3660) |
| **Birth Length (cm)** | 2826 | 49 (47.5 to 50.5) |
| **Gestation (wks.)** | 2862 | 39.5 (38.2 to 40.7) |
| **Weight (kg) at 17 years** | 1251 | 65.4 (58.2 to 74.6) |
| **Height (cm) at 17 years** | 1251 | 172 (165 to 179) |
| **BMI (kg/m2) at 17 years** | 1251 | 22.06 (19.96 to 24.33) |
| **Abdominal skinfold thickness (mm) at 17 years** | 1155 | 19.9 (12.6 to 27.5) |
| **Triceps Skinfold Thickness (mm) at 17 years** | 1170 | 14.0(9.0 to 19.5) |

**Supplementary Table 1 Characteristics of the RAINE study participants.**

**Supplementary Table 2 Genomic coordinates for CpGs measured in *CDKN2A***

|  | **Base pairs from TSS** |  |
| --- | --- | --- |
| **CpG** | **ANRIL** | **Coordinates (Hg19)** |
| 1 | -1069 | chr9: 21993721 |
| 2 | -1093 | chr9: 21993697 |
| 3 | -1096 | chr9: 21993694 |
| 4 | -1136 | chr9: 21993654 |
| 5 | -1145 | chr9: 21993645 |
| 6 | -1152 | chr9: 21993638 |
| 7 | -1161 | chr9: 21993629 |
| 8 | -1187 | chr9: 21993603 |
| 9 | -1207 | chr9: 21993583 |

**Supplementary Table 3 Association between *CDKN2A* CpG methylation and cellular type in the RAINE cohort**

| **CpG** | **N** | **CD8 T cells** | | **CD4 T cells** | | **NK cells** | | **B cells** | | **Monocytes** | | **Granulocytes** | |
| --- | --- | --- | --- | --- | --- | --- | --- | --- | --- | --- | --- | --- | --- |
|  |  | **Pearson Correlation** | **Significance (2-tailed)** | **Pearson Correlation** | **Significance (2-tailed)** | **Pearson Correlation** | **Significance (2-tailed)** | **Pearson Correlation** | **Significance (2-tailed)** | **Pearson Correlation** | **Significance (2-tailed)** | **Pearson Correlation** | **Significance (2-tailed)** |
| **1** | 1042 | 0.431 | <2.2e-16 | 0.4 | < 2.2e-16 | 0.221 | 6.45e-13 | 0.141 | 4.29e-06 | -0.314 | < 2.2e-16 | -0.529 | < 2.2e-16 |
| **2** | 1013 | 0.359 | <2.2e-16 | 0.3 | < 2.2e-16 | 0.252 | 4.44e-16 | 0.085 | 0.0104 | -0.229 | 2.41e-12 | -0.452 | < 2.2e-16 |
| **3** | 969 | 0.443 | <2.2e-16 | 0.411 | < 2.2e-16 | 0.253 | 1.33e-15 | 0.142 | 9.50e-06 | -0.319 | < 2.2e-16 | -0.562 | < 2.2e-16 |
| **4** | 1081 | 0.611 | <2.2e-16 | 0.553 | < 2.2e-16 | 0.371 | < 2.2e-16 | 0.215 | 1.36e-12 | -0.402 | < 2.2e-16 | -0.783 | < 2.2e-16 |
| **5** | 1056 | 0.596 | <2.2e-16 | 0.508 | < 2.2e-16 | 0.396 | < 2.2e-16 | 0.21 | 7.89e-12 | -0.386 | < 2.2e-16 | -0.757 | < 2.2e-16 |
| **6** | 1040 | 0.607 | <2.2e-16 | 0.583 | < 2.2e-16 | 0.381 | < 2.2e-16 | 0.238 | 1.31e-14 | -0.427 | < 2.2e-16 | -0.804 | < 2.2e-16 |
| **7** | 986 | 0.618 | <2.2e-16 | 0.496 | < 2.2e-16 | 0.42 | < 2.2e-16 | 0.185 | 8.90e-09 | -0.394 | < 2.2e-16 | -0.762 | < 2.2e-16 |
| **8** | 1070 | 0.615 | <2.2e-16 | 0.643 | < 2.2e-16 | 0.351 | < 2.2e-16 | 0.248 | 4.44e-16 | -0.432 | < 2.2e-16 | -0.848 | < 2.2e-16 |
| **9** | 1009 | 0.591 | <2.2e-16 | 0.621 | < 2.2e-16 | 0.364 | < 2.2e-16 | 0.243 | 8.88e-15 | -0.441 | < 2.2e-16 | -0.824 | < 2.2e-16 |

**Supplementary Table 4 Variance Inflation Factors (VIFs) for all coefficients in regression models with Log_e_ BMI as outcome**

|  | **Sex** | **Age** | **CpG** | **CD8 T cells** | **CD4 T cells** | **NK cells** | **B cells** | **Monocytes** | **Granulocytes** |
| --- | --- | --- | --- | --- | --- | --- | --- | --- | --- |
|  |  |  | **CpG1** |  |  |  |  |  |  |
| **Model 1** | 1.18 | 1.02 | 1.44 | 25.21 | 61.32 | 27.61 | 14.25 | 11.36 | 110.47 |
| **without Granulocytes** | 1.17 | 1.01 | 1.44 | 1.36 | 1.53 | 1.30 | 1.32 | 1.49 | NA |
|  |  |  | **CpG2** |  |  |  |  |  |  |
| **Model 2** | 1.19 | 1.03 | 1.29 | 25.44 | 61.06 | 28.34 | 14.49 | 11.59 | 112.07 |
| **without Granulocytes** | 1.18 | 1.01 | 1.29 | 1.33 | 1.48 | 1.30 | 1.34 | 1.50 | NA |
|  |  |  | **CpG3** |  |  |  |  |  |  |
| **Model 3** | 1.19 | 1.03 | 1.57 | 25.56 | 61.52 | 27.93 | 14.57 | 11.58 | 112.77 |
| **without Granulocytes** | 1.19 | 1.02 | 1.56 | 1.38 | 1.57 | 1.35 | 1.33 | 1.49 | NA |
|  |  |  | **CpG4** |  |  |  |  |  |  |
| **Model 4** | 1.20 | 1.03 | 3.17 | 24.95 | 61.21 | 30.26 | 14.67 | 11.32 | 113.71 |
| **without Granulocytes** | 1.19 | 1.02 | 3.13 | 1.83 | 2.09 | 2.02 | 1.33 | 1.50 | NA |
|  |  |  | **CpG5** |  |  |  |  |  |  |
| **Model 5** | 1.19 | 1.03 | 3.03 | 25.19 | 61.71 | 29.92 | 14.51 | 11.37 | 115.73 |
| **without Granulocytes** | 1.19 | 1.02 | 2.96 | 1.82 | 1.93 | 2.02 | 1.34 | 1.49 | NA |
|  |  |  | **CpG6** |  |  |  |  |  |  |
| **Model 6** | 1.20 | 1.03 | 3.50 | 24.94 | 61.33 | 29.84 | 14.49 | 11.40 | 114.82 |
| **without Granulocytes** | 1.19 | 1.02 | 3.43 | 1.80 | 2.21 | 2.11 | 1.33 | 1.50 | NA |
|  |  |  | **CpG7** |  |  |  |  |  |  |
| **Model 7** | 1.20 | 1.03 | 3.29 | 25.32 | 62.95 | 30.34 | 15.07 | 11.82 | 116.86 |
| **without Granulocytes** | 1.19 | 1.02 | 3.21 | 1.91 | 1.96 | 2.16 | 1.33 | 1.50 | NA |
|  |  |  | **CpG8** |  |  |  |  |  |  |
| **Model 8** | 1.20 | 1.04 | 4.67 | 24.80 | 61.36 | 30.10 | 14.65 | 11.38 | 114.09 |
| **without Granulocytes** | 1.19 | 1.03 | 4.50 | 2.03 | 2.89 | 2.35 | 1.33 | 1.49 | NA |
|  |  |  | **CpG9** |  |  |  |  |  |  |
| **Model 9** | 1.19 | 1.04 | 3.91 | 24.65 | 61.09 | 29.64 | 14.23 | 11.32 | 112.86 |
| **without Granulocytes** | 1.17 | 1.03 | 3.82 | 1.78 | 2.50 | 2.20 | 1.34 | 1.49 | NA |

**Supplementary Table 5 Association between Log_e_ BMI and cellular type in the RAINE cohort**

|  | | **CD8 T cells** | **CD4 T cells** | **NK cells** | **B cells** | **Monocytes** | **Granulocytes** |
| --- | --- | --- | --- | --- | --- | --- | --- |
| log_e_BMI17 | Pearson Correlation | -.098^**^ | -.082^*^ | -.079^*^ | -.079^*^ | 0.043 | .160^**^ |
|  | Significance (2-tailed) | 0.003 | 0.015 | 0.018 | 0.018 | 0.201 | <0.001 |
|  | N | 894 | 894 | 894 | 894 | 894 | 894 |
